# Differential Neurobiological Markers in Phenotype-stratified Rats Modeling High or Low Vulnerability to Compulsive Behavior: A Narrative Review

**DOI:** 10.2174/1570159X21666221121091454

**Published:** 2023-07-10

**Authors:** Elena Martín-González, Manuela Olmedo-Córdoba, Pilar Flores, Margarita Moreno-Montoya

**Affiliations:** 1 Department of Psychology and Health Research Center (CEINSA), University of Almería, Almeria, Spain

**Keywords:** Compulsive behavior, schedule-induced polydipsia, serotonin 5-HT2A receptor, obsessive-compulsive disorder, animal model, endophenotype

## Abstract

Compulsivity is a key manifestation of inhibitory control deficit and a cardinal symptom in different neuropsychopathological disorders such as obsessive-compulsive disorder, schizophrenia, addiction, and attention-deficit hyperactivity disorder. Schedule-induced polydipsia (SIP), is an animal model to study compulsivity. In this procedure, rodents develop excessive and persistent drinking behavior under different food-reinforcement schedules, that are not related to homeostatic or regulatory requirements. However, there are important individual differences that support the role of high-drinker HD rats as a compulsive phenotype, characterized in different paradigms by inhibitory response deficit, cognitive inflexibility, and resistant to extinction behavior; with significant differences in response to pharmacological challenges, and relevant neurobiological alterations in comparison with the control group, the non-compulsive low drinker LD group on SIP. The purpose of this review is to collate and update the main findings on the neurobiological bases of compulsivity using the SIP model. Specifically, we reviewed preclinical studies on SIP, that have assessed the effects of serotonergic, dopaminergic, and glutamatergic drugs; leading to the description of the neurobiological markers, such as the key role of the serotonin 5-HT2A receptor and glutamatergic signaling in a phenotype vulnerable to compulsivity as high drinker HD rats selected by SIP. The review of the main findings of HD rats on SIP helps in the characterization of the preclinical compulsive phenotype, disentangles the underlying neurobiological, and points toward genetic hallmarks concerning the vulnerability to compulsivity.

## 1 INTRODUCTION

Compulsivity is characterized by persistent, inflexible, and excessive behaviors, either as a way to avoid perceived adverse consequences or as dependent on rigid rules; being a key factor in the loss of control over behavior [1, 2]. Compulsive behaviors are the cardinal symptom of obsessive-compulsive disorder (OCD), which affects 2-3% of the population and is considered a chronic and disabling condition [3, 4]. Moreover, compulsivity is also present in many neuropsychiatric disorders such as pathological gambling, substance abuse, addiction, and eating disorders [5]. Therefore, compulsivity has been considered a transdiagnostic trait that should be studied according to a dimensional approach [2], according to the U.S. National Institute of Mental Health (NIMH) Research Domain Criteria RDoC [6] and the Roadmap for Mental Health Research in Europe ROAMER [7].

The research on preclinical models might help scientists and clinicians to improve their knowledge about the possible identification of biomarkers in the compulsive phenotype, creating new mechanisms for the prevention and treatment of psychopathological disorders related to compulsivity. Schedule-induced polydipsia is a preclinical model that has been used to study compulsivity. Polydipsia induced in rodents by exposure to a program of intermittent food reinforcement was a phenomenon discovered in the sixties by John Falk [8]. This author observed that food-deprived rats exposed to fixed-time FT intervals of food pellet reinforcement developed an adjunctive behavior of excessive drinking to a bottle of water available throughout the experimental session. the amount of water consumed during the session exceeded the regulatory water intake [9, 10]. Thus, SIP is a procedure that results in the development of excessive, persistent, and maladaptive drinking behavior in rodents exposed to FT schedules. In 2012, two of the authors of the present manuscript, Moreno and Flores, reviewed the validity criteria of SIP as a preclinical model for the study of compulsivity [11]. In each of the criteria, we can highlight the following: 1. Face validity, SIP induces a persistent and excessive drinking behavior, and does not depend on physiological needs but on situational distress created by intermittent reinforcement [8, 10]. These are behavioral characteristics of compulsive behavior according to DSM-V. In fact, in humans, there is an analogous phenomenon called psychogenic polydipsia, defined as non-regulatory excessive drinking. This behavior is present in 6-20% of patients with schizophrenia and compulsive spectrum disorders [12-15]; 2. Construct validity, the neurobiological basis of SIP behavior is related to compulsivity: the underlying neuroanatomical circuit on SIP involves areas such as the prefrontal cortex, hippocampus, and nucleus accumbens [16-19]; there is a relevant implication of the neuroendocrine function of the hypothalamus-pituitary-adrenal (HPA) axis in SIP behavior [18, 20]; there is an important neurochemical basis under SIP by serotonin, 5-HT receptors and dopamine signaling [17-19, 21-23]; 3. Predictive validity has been demonstrated by the reduction of compulsive drinking in SIP, without affecting regulatory drinking, by drugs used in the treatment of OCD symptomatology, including antipsychotics and selective serotonin reuptake inhibitors (SSRIs) [24-27]; 4. Reliability, the compulsive behavior in SIP is easily measurable, stable in all individuals, and reproducible in any laboratory under a specific set of conditions. This favors the extension of the model, comparison, and progress in research on this behavior. Therefore, SIP has been considered a valid model of compulsive behavior as it meets the criteria of face validity, construct validity, predictive validity, and reliability [11].

In a previous review [11], we collated the main findings using antipsychotics and serotonergic drugs on SIP, showing its pharmacological efficacy and pointing towards the neurochemical underlying mechanisms of compulsivity. In the present review, our goal was to update the results on the pharmacological modulation of compulsivity using SIP, by further analysis in preclinical studies that have assessed the effects of different drugs on SIP, leading to the description of the neurobiological markers in a phenotype vulnerable to compulsivity as high drinker HD rats on SIP. We consider findings in brain biomarkers of highly compulsive rats selected by SIP concerning those observed in OCD and in other neuropsychopathological disorders that have compulsivity as the main relevant symptom. For this purpose, the rationale was to include articles that were: (1) published in English, (2) on the compulsive phenotype of SIP in rodents, (3) related to psychopharmacology, neurochemical activity, and/or brain morphology, and (4) published after 2012, because of our previous review.

## 2 THE COMPULSIVE PHENOTYPE SELECTED BY SCHEDULE-INDUCED POLYDIPSIA

SIP is a preclinical model of compulsivity that has been used to study the behavioral, neurochemical, and neurobiological characteristics associated with different disorders that share compulsive behaviors such as OCD, schizophrenia, and alcohol abuse [28-40]. However, there are important individual differences in SIP. Not all food-deprived animals develop adjunctive drinking under an intermittent reinforcement schedule, showing clear intergroup differences after 15-20 sessions on SIP [11]. According to SIP acquisition, two populations might be selected: high drinker (HD) rats considered compulsive *versus* low drinker (LD) rats considered non-compulsive; based on whether their drinking rates (mean for each rat over the last 3-5 sessions) were above or below the group median. The identification of a compulsive group, the HD animals by the SIP model, allows us to study the associated behavioral characteristics, as well as neuroanatomical and neurochemical aspects.

In the characterization of the behavioral phenotype, animals with an increased drinking behavior on SIP have revealed a compulsive phenotype demonstrated by paradigms that measure different aspects of inhibitory control behavior. The HD animals selected by SIP, as well as the Spontaneously Hypertensive rats SHR and Roman High Avoidance RHA rats, which also exhibit a high rate of compulsive drinking on SIP, have shown increased cognitive impulsivity in the Delay-discounting task [23, 41-43]. Moreover, HD animals and RHA rats showed increased motor impulsivity measured by premature responses on the sustained attentional 5-Choice Serial Reaction Time task 5-CSRTT [23, 42]. Regarding other aspects of inhibitory control dysfunctions related to compulsivity, HD showed attentional deficit by reduced latent inhibition compared to low-drinker LD rats [39]. However, one of the most important aspects regarding the compulsive phenotype has been the consistent results on cognitive inflexibility and resistance to extinction behaviors in HD compared to LD rats selected by SIP. Thus, different paradigms have demonstrated an increased cognitive inflexibility by an increase in the trials to criterion, the number of perseverative responses, or errors in different versions of the Reversal learning task [39, 45, 46]. Indeed, this behavioral inflexibility has also been evidenced by a resistance to extinction behavior in the HD rats, characterized by an increased compulsivity assessed by perseverative responses under an extinction condition on the 5-CSRT task [42]; insensibility to outcome devaluation during extinction under selective satiation [45]; increased fear memory on Fear Condition task [47] and a resistant emotional memory on Passive Avoidance task compared to LD rats [48].

According to this compulsive behavioral phenotype, the study on the selection of animals by highly compulsive drinking on SIP has also revealed neuroanatomic and neurochemical markers. In this sense, pharmacological studies have shown SIP to be a useful model to assess the modulation of compulsivity and have a better knowledge of the underlying mechanisms.

## 3 NEUROCHEMICAL BIOMARKERS ON SIP

### 3.1 Serotonergic Biomarkers

The contribution of serotoninergic drugs to unveil the contribution of the neurobiological mechanisms in high-compulsive drinking on SIP is summarized in Table **1**. According to the literature, serotoninergic neurochemistry seems to be a relevant biomarker in compulsive drinking on SIP. In 1993, Woods *et al*. [49], tested the chronic administration of fluoxetine and clomipramine on SIP reduction, assessing the efficacy of SSRIs in this preclinical model and pointing toward its utility as a research tool for new drugs in the clinical treatment of OCD patients. These results have been replicated and expanded by the reduction of drinking behavior on SIP by the 5-HT-norepinephrine reuptake inhibitor duloxetine [50]. Furthermore, a review by Platt *et al.* [26] and Moreno and Flores [11] collected preclinical data for serotonergic drugs such as serotonin 5-HT6 and 5-HT2C receptor agonists that have been shown to decrease compulsive drinking on SIP [11, 26]. Specifically, different studies have demonstrated the key role of serotonin 5-HT2C receptor on SIP; demonstrated that its agonists reduced compulsive drinking behavior on SIP and this effect was reversed by serotonin 5-HT2C antagonists [51-53]. Thus, supporting the role of 5-HT2C receptors in the efficacy of SSRIs to reduce compulsivity.

However, as the SIP procedure is sensitive to individual differences, recent studies on high-compulsive rats selected by SIP have posited new serotonergic biomarkers related to clinical data on OCD and compulsive neuropsychopathological disorders. In this sense, a recent study with the serotonin 5-HT2C agonist Locaserin has replicated the observed reduction in compulsive drinking in high-drinker HD rats selected by SIP, showing a specific effect after a stress condition [54]. Moreover, HD rats have shown a dose-dependent reduction in compulsive drinking after the systemic administration of the SSRI citalopram and the serotonin 5-HT2A/C agonist DOI [55]. This study suggested the key role of serotonin 5-HT2A/C receptor function associated with the presumed compulsive trait of HD rats selected by SIP. Moreover, the combination of the serotonin 5-HT2A/C agonist DOI with specific 5-HT2A antagonist Kentaserin and M100907, as well as, the specific 5-HT2C antagonist SB242084, highlighted the specific contribution of serotonin 5-HT2A receptors in the inhibition of the compulsive drinking behavior in HD rats on SIP. Thus, the combined administration of ketanserin or M100907 blocked the reduction in compulsive drinking induced by DOI in HD rats on SIP, whereas SB242084 did not.

The role of the serotonin 5-HT2A receptor as a possible biomarker of vulnerability to compulsion was further investigated in the compulsive HD rats selected by SIP through the assessment of its expression, function, and neurochemistry in the brain. Compulsive HD rats selected by SIP revealed a specific reduction of the serotonin 5-HT2A receptor binding in the frontal cortex and basolateral amygdala compared to the no-compulsive LD rats [56, 57]. Moreover, these results have been recently supported by a reduction in the 5-HT2A gene expression in the frontal cortex of HD compared to LD rats selected by SIP [58]. Clinical studies have pointed out the relevance of the serotonin 5-HT2A receptor as a possible hallmark of vulnerability in OCD, for example: first, in a meta-analysis of genetic association studies OCD vulnerability was associated with genetic polymorphism in this receptor [59]; second, neuroimaging studies have observed a reduced availability in the frontal cortex in drug-naïve OCD patients [60] and third, it has been suggested a negative correlation between the reduced serotonin 5-HT2A receptor and the clinical OCD severity as well as the age of onset [61]. Preclinical studies have also revealed a reduction in serotonin 5-HT2A receptor availability in the frontal cortex of compulsive dogs [62] and rats selected according to high cognitive inflexibility in a reversal-learning task in the orbitofrontal cortex [63]. Furthermore, the study of Mora *et al.* [56] replicated and expanded the results observed by Navarro *et al.* [55]. Therefore, the specific contribution of frontal 5-HT2A receptors in the compulsive behavior of HD rats on SIP was also confirmed by the reduction of compulsive drinking after the microinfusion of the 5-HT2A/C agonist DOI into the medial prefrontal cortex; an effect that was reversed by its combination with the serotonin 5-HT2A receptor antagonist M100907 and not by the 5-HT2C antagonist SB242084.

The serotonergic dysfunction of compulsive HD rats selected by SIP has also been evidenced by an increased serotonin activity in the amygdala [44] and the frontal cortex [56]. Although these results could be considered counterintuitive, according to the effects of fluoxetine and citalopram on SIP, they might reflect possible compensatory mechanisms that would also be related to the reduction in the 5-HT2A receptor binding. In this sense, a microdialysis experiment revealed that the reduction in compulsive drinking of HD after DOI administration might be mediated through an increase of serotonin but also by its action in the restoration of glutamatergic mechanisms [56]. New pharmacological studies have explored the role of glutamate in compulsive HD rats on SIP [47].

Moreover, the decrease in serotonergic signaling by a tryptophan TRP depletion diet revealed an enhancement in compulsive drinking in HD on SIP, this effect was not observed in LD nor in Lister Hooded rats that have no acquisition of SIP [64]. This points toward the involvement of a sensitive dysfunctional serotonergic system in the compulsive phenotype of HD rats, that might be modulated through diet according to the gut-brain axis. The gut microbiota regulates TRP metabolism and may affect global serotonin synthesis in the enteric and central nervous systems, suggesting a possible involvement of gut microbiota in compulsive spectrum disorders [65, 66]. The compulsive HD rats have revealed a reduced microbial diversity than LD rats, irrespective of the diet. Furthermore, the exposure to a TRP depletion diet, that effectively reduced peripheral plasma serotonin levels in both HD and LD rats, revealed a reduction of bacterial evenness and a highly functionally organized community only in HD TRP depleted rats [67]. This finding supports the hypothesis of the compulsive phenotype of HD rats as a population with a more fragile bacterial community to external changes due to the dominance of a low number of species in the gut. The reduced microbial diversity and its impact on the serotonergic system modulating the gut-brain axis might be a relevant biomarker in compulsive spectrum disorders.

### 3.2 Dopamine and Other Monoamine Biomarkers

Dopamine has a relevant role in compulsive drinking on SIP, its contribution in the last decade has been summarized in Table **2**. Classical studies demonstrated the efficacy of typical and atypical antipsychotics to reduce compulsive drinking on SIP [11]. D-amphetamine and Cocaine, which enhance dopamine signaling, have been shown to reduce dose-dependent compulsive drinking in HD rats on SIP compared to LD rats [68]. Curiously, a dose-response study with methamphetamine, a monoamine transmission facilitator that inhibits the dopamine transporter involved in its reuptake, revealed an inverted U effect in HD and LD rats on SIP, in which low doses increased and higher doses reduced compulsive drinking in both groups [69]. Moreover, the Spontaneously Hypertensive rats SHR, which are characterized by a high rate of compulsive drinking on SIP, have demonstrated a different response to acute challenge with D-amphetamine and dopamine D1 and D2 receptor agonists and antagonists compared to control rats [70]. SHRs were less sensitive to the reduction in rates of drinking by dopaminergic agents than control rats. In this sense, the individual differences in SIP have also been related to alterations in dopamine D1 and D2 receptor binding in mesolimbic areas [71], in which HD showed a higher binding for D2 receptors and lower binding for D1 receptors compared to LD rats. Moreover, the induction of hyperactivity on the dopamine system by subchronic treatment with D-amphetamine induced a sensitization response enhancing compulsive drinking on SIP [35, 72]. Thus, supporting the DA dysfunction or aberrant activity of other circuits that modulate DA activity in animals with a high rate of compulsive drinking on SIP. Indeed, a recent study with a novel pharmacological target, the Trace Amine Associated Receptor 1 (TAAR1), which plays a modulatory role in the dopaminergic system, demonstrated a reduction in compulsive drinking with a wide range of doses of the highly selective partial TAAR1 agonist RO5263397 [73].

However, the modulation of other monoamines such as noradrenaline according to a possible hyperactivity dysfunction, did not shed clear effects. The acute administration of atomoxetine did not induce any changes in HD rats in compulsive drinking on SIP [54, 55]. Furthermore, the subchronic administration of methylphenidate appears to reduce SIP in control rats but not in SHR rats, pointing towards that the therapeutic effects of methylphenidate on compulsive drinking on SIP require higher doses in SHR relative to control strains [74].

### 3.3 Glutamate Biomarkers

The contribution of glutamatergic drugs to the knowledge of the neurobiological mechanisms in high compulsive drinking on SIP is summarized in Table **3**. Recent studies suggest glutamate-modulating drugs as a possible psychophysiological strategy for reducing compulsive symptoms [75]. In the last decade, there has been a growing interest in research on the implication of the glutamatergic system on compulsivity. One of the first studies that explored the implication of glutamate on SIP was by Hawken *et al.* [76], where the subchronic treatment with the N-methyl-D-aspartate NMDA receptor antagonist MK-801 significantly increased SIP.

The acute administration of glutamatergic drugs in HD and LD rats has revealed no conclusive effects regarding the role of glutamate in compulsive drinking on SIP. Thus, the NMDA receptor antagonist Ketamine did not induce any effects in compulsive drinking on SIP [69]. However, other compounds that are being used in clinical studies to treat SRI-resistant OCD patients [75] have significatively reduced compulsive drinking on SIP. Memantine, an uncompetitive receptor NMDA antagonist, and Lamotrigine, with inhibitory action on the excitatory amino acids, reduced compulsive drinking in HD compared to LD rats on SIP; while N-Acetylcysteine a glutathione precursor did not have any effect [47]. Moreover, the study on the neurobiological underlying brain mechanisms has not shed much light on the relation between glutamatergic receptors and compulsive drinking on SIP. Thus, the assessment of the glutamatergic mGlu receptor binding in the frontal cortex as well as the administration of the glutamate receptor mGlu2/3 LY379268 agonist did not show any differences between HD and LD rats selected by SIP [56].

Nevertheless, the assessment of the neurochemical activity in basal conditions by microdialysis in the medial prefrontal cortex showed a reduced glutamatergic signal in the compulsive HD rats selected by SIP [56]. Moreover, the therapeutic effect of the administration of the serotonin 5-HT2A/C receptor agonist DOI on the reduction of compulsive drinking in HD rats on SIP seems to be mediated through a restoration in the glutamatergic signal in the medial prefrontal cortex. The dysfunction in the glutamatergic function might induce relevant neuroplastic changes in the brain that can affect other neurobiological and chemical systems. Finally, the recent finding of a hyperlipidemic, hypoglycemic, and hyperglutaminergic profile in serum samples of HD compared to LD rats reveals interesting peripheral markers of the possible neurobiological alterations in the compulsive phenotype [77]. Thus, pointing toward the possible implication of dietary and metabolic factors underpinning the vulnerability to compulsive behaviors.

## 4 ACTIVITY AND MORPHOLOGICAL MARKERS IN THE BRAIN ON SIP

Several studies have revealed neurobiological differences related to changes in brain structures between compulsive HD rats and LD selected by SIP (Table **4**).

Regarding brain activity, HD animals showed an elevated c-Fos protein quantification in the medial prefrontal cortex [71]. In accordance with these results, other laboratories also demonstrated that rats with SIP acquisition had increased FosB/ΔFosB expression in the medial prefrontal cortex, and lateral and ventral orbitofrontal cortex [34]. Moreover, the selection of a specific population with a high drinking profile on SIP through cluster analyses, the Compulsive Drinker CD group, also showed an elevated c-fos count pointing towards hyperactivity in the lateral orbitofrontal cortex relative to the LD group, with a strong positive correlation between this brain area and the frequency of licking [78]. The same study showed a trend toward significance in the basolateral amygdala, in CD compared with LD rats, indicating hyperactivity in this region [78].

Related to brain morphology, the assessment of volume in post-mortem brain samples using the stereology technique, revealed that HD rats showed a larger volume of the BLA and reduced volume of dorsal Hippocampus compared to LD rats [57]. However, no differences between HD and LD were found in volume either in the prelimbic or infralimbic cortex [57]. HD compulsive rats also showed brain changes related to neuroplasticity, indicating a possible neuroplastic mechanism underlying compulsive drinking on SIP. Exposure to 20 sessions of SIP led to an increase in dendritic spine density in dorsolateral Striatum neurons, which was highly correlated with the level of drinking acquisition on SIP [79]. However, there were no differences either in dendritic spine density or in the morphological structure of the dendrites of the anterior PFC in SIP animals relative to their control counterparts [79].

Finally, regarding the white matter, the assessment of myelin in post-mortem brain samples through analyzing Myelin basic protein we found that HD rats showed less myelination in the center of the Corpus Callosum, Striatum, and BLA compared with LD rats [39].

## CONCLUSION

The main findings regarding the compulsive phenotype of high-drinking rats on SIP highlight the contribution of serotoninergic and dopaminergic dysfunction through neuropharmacological studies. The possible genetic markers of this preclinical model of compulsivity point towards a dysregulation in dopamine D1 and D2 receptors, as well as a reduction in serotonin 5-HT2A receptors. However, these neurobiological alterations could also modulate other neurochemical systems such as the glutamatergic signaling pathways. Therefore, central and peripheral measurements have shown an alteration in glutamate levels in compulsive HD compared to LD rats selected by SIP. Moreover, the results reviewed support the hypothesis of the compulsive phenotype of HD rats as a population more fragile to external changes, such as stress or diet, due to the dominance of a low number of species in the gut. The altered glutamatergic signaling in compulsive HD rats might also have a key role in the neuroplasticity observed in HD rats, according to the differences in brain volume and myelination. However, future studies should disentangle how these changes impact the prefrontal cortex function, as revealed by its altered activity in compulsive high drinker rats on SIP. Moreover, future lines of research using the compulsive phenotype on SIP might help for example 1) detect environmental conditions or external changes that trigger the development of the compulsive phenotype, 2) study the neuroplasticity mechanisms under the development of compulsive behavior, and 3) perform proof of concept studies to validate new potential pharmacological targets for the treatment of compulsive spectrum disorders. These proposals might help in early detection and the generation of new treatments, as well as in an improvement of new indicators to evaluate therapeutic results of pharmacological, neuromodulation, and behavioral treatments on compulsivity. Thus, the research on the compulsive phenotype of rats selected by SIP could provide additional insight for potential neurobiological hallmarks and new treatments on the vulnerability to compulsive behavior, characteristic of OCD and other neuropsychopathological disorders.

## Figures and Tables

**Table 1:** Serotonergic biomarkers on SIP.

**Treatment**	**SIP Results**	**Biomarkers Results**	**References**
**-**	**-**	HD↑ 5-HT activity in amygdala & FC *vs.* LD	[44, 56]
Fluoxetine & clomipramine (chronic) *SSRIs*	↓ Compulsive drinking	*-*	[49]
Duloxetine (subchronic) *5-HT-norepinephrine reuptake inhibitor*	↓ Drinking behavior	*-*	[50]
SB-242084 (acute) *5-HT2C antagonists*	↑ Compulsive drinking	*-*	[51]
CPD1 (chronic) *5-HT2C agonist*	↓ Compulsive drinking	*-*	[52]
WAY-163909 (acute) *5-HT2C agonist*	↓ Compulsive drinking	*-*	[53]
Locaserin *5-HT2C agonist*	↓ Compulsive drinking but ↑↑ Effect specific after stress condition (HD>LD)	*-*	[54]
Citalopram (acute) *SSRIs* DOI (acute) *5-HT2A/C agonist*	HD ↓compulsive drinking *vs.* LD (dose-dependent)	-	[55]
DOI + *5-HT2A antagonist* DOI + Ketanserin DOI + M100907 DOI *+5-HT2C antagonist* { DOI + SB 242084	Ketanserin & M100907 blocked effect DOI (HD) SB 242084 × blocked effect DOI (HD)		
DOI (5-HT2A/C agonist)	Microinfusion of DOI in mPFC ↓ compulsive drinking	-	[56]
DOI + *5-HT2A antagonist* { DOI + M100907 DOI + *5-HT2C antagonist* { DOI + SB242084	M100907 blocked effect DOI (HD) SB 242084 × blocked effect DOI (HD)		
		HD ↓serotonin 5-HT2A receptor binding in FC & BLA *vs.* LD (basal)	[56, 57]
		HD ↓5-HT2A gene expression in FC *vs.* LD	[58]
**-**	**-**	Dogs compulsives ↓ serotonin 5-HT2A receptor	[62]
-	-	High inflexivility rats by RL task ↓ serotonin 5-HT2A receptor binding in OFC	[63]
-	-	HD↑ 5-HT activity in amygdala & FC *vs.* LD	[44, 56]
TRP depletion diet	↑Compulsive drinking in HD × Effect in LD & LH	-	[64]
TRP depletion diet	-	HD ↓microbial diversity *vs.* LD (irrespective of diet). Diet ↓peripheral 5-HT levels in HD & LD Diet ↓bacterial evenness & functionally organized community in HD	[67]

**Abbreviations**: increase (↑); decrease (↓); same (=); and (&); *versus* (*vs.*); no/none (X); higher (>); high drinker (HD); low drinker (LD); lister Hooded (LH); frontal cortex (FC); medial prefrontal cortex (mPFC); basolateral amygdala (BLA); orbitofrontal cortex (OFC); tryptophan (TRP); 5-HT2C receptor agonist ((3S)-3-Methyl-1-[4-(trifluoromethyl)-7-benzofuranyl]-piperazine) (CPD1); selective serotonin reuptake inhibitors (SSRIs); serotonin (5-HT); serotonin 2A receptors (5-HT2A); serotonin 2C receptors (5-HT2C); reversal learning task (RL).

**Table 2:** Dopamine and other monoamine biomarkers on SIP.

**Treatment**	**SIP Results**	**Biomarkers Results**	**References**
**Dopamine**			
D-amphetamine (subchronic)	Sensitization response ↑ compulsive drinking	-	[35, 72]
D-amphetamine & cocaine (acute)	HD ↓compulsive drinking *vs.* LD (dose-dependent)	-	[68]
Methamphetamine (acute)	Low doses ↑ compulsive drinking Higher doses ↓ compulsive drinking (HD & LD)	-	[69]
D-amphetamine, eticlopride, quinpirole, SCH 23390, SKF 38393 (acute)	SHR ↓sensitive to the reduction in rates of drinking *vs.* control group	-	[70]
-	-	HD ↑binding D2 receptors *vs.* LD HD ↓binding D1 receptors *vs.* LD	[71]
RO5263397 (acute & subchronic) *selective partial TAAR1 agonist*	↓Compulsive drinking (various doses)	-	[73]
**Noradrenaline**			
Atomoxetine (acute) *selective norepinephrine reuptake inhibitor*	Effect in compulsive drinking (HD)	-	[54, 55]
Methylphenidate (subchronic)	Effect in compulsive drinking in SHR but ✓ effect in control group	-	[74]

**Abbreviations**: Increase (↑); decrease (↓); same (=); and (&); *versus* (*vs.*); no/none (X); yes (✓); high drinker (HD); low drinker (LD); spontaneously hypertensive rat (SHR); dopamine receptor D1 (D1); dopamine receptor D2 (D2).

**Table 3:** Glutamate biomarkers on SIP.

**Treatment**	**SIP Results**	**Biomarkers Results**	**References**
Memantine (acute) *uncompetitive NMDA * *antagonist* Lamotrigine (acute) amino acids inhibitor	HD ↓ compulsive drinking *vs.* LD	-	[47]
NAC (acute)	X Effect in compulsive drinking		
LY379268 (acute) mGlu2/3 agonist	X Effect in compulsive drinking	HD LD mGlu2/3 receptor in FC (basal) HD ↓ glutamatergic signal *vs.* LD in mPFC (basal)	[56]
Ketamine (acute) *NMDA antagonist*	X Effect in compulsive drinking	-	[69]
MK-801 (subchronic) *NMDA antagonist*	↑SIP acquisition	-	[76]
-	-	HD present hyperlipidemic, hypoglycemic & hyperglutaminergic profile *vs.* LD	[77]

**Abbreviations**: Increase (↑) ; decrease (↓); same (=); and (&); *versus* (*VS.*); no/none (X); high drinker (HD); low drinker (LD); N-Acetylcysteine (NAC); frontal cortex (FC); medial prefrontal cortex (mPFC); N-Methyl-D-aspartate (NMDA).

**Table 4:** Activity and morphological markers in the brain on SIP.

**Types**	**Areas**	**Results**	**Others (SIP)**	**References**
Brain activity	mPFC, VLOFC & LOFC	SIP acquisition ↑ FosB/ΔfosB expression *vs.* non-SIP	-	[34]
	mPFC	HD ↑c-Fos protein *vs.* LD	-	[71]
	LOFC & BLA	CD ↑c-Fos *vs.* LD	↑c-Fos LOFC ↑ frequency licking (correlation)	[78]
White matter	Corpus Callosum Sriatum and BLA	HD ↓ myelination *vs.* LD	-	[39]
Brain morphology	BLA	HD ↑ volume *vs.* LD	-	[57]
	Dorsal HC	HD ↓ volume *vs.* LD		
	Prelimbic & infralimbic cortex	HD LD volume		
Neuroplasticity	DLS	SIP ↑dendritic spine density *vs.* control	↑SIP acquisition ↑ dendritic spine density (correlation)	[79]
	aPFC	SIP control spine density & structural dendrites	-	

**Abbreviations**: Increase (↑) ; decrease (↓); same (=); and (&); *versus* (*VS.*); high drinker (HD); low drinker (LD); compulsive drinker (CD); medial prefrontal cortex (mPFC); ventral lateral orbitofrontal (VLOFC); lateral orbitofrontal (LOFC); basolateral amygdala (BLA); hippocampus (HC); dorsolateral striatum (DLS); anterior prefrontal cortex (aPFC); schedule-induced polydipsia (SIP).

## LIST OF ABBREVIATIONS

HD: High Drinker
HPA: Hypothalamus-pituitary-adrenal
LD: Low Drinker
OCD: Obsessive-compulsive Disorder
